# Diagnostic accuracy of non-contrast magnetic resonance angiogram of infra-popliteal arteries prior to fibular-free flap harvest

**DOI:** 10.1186/1532-429X-18-S1-P354

**Published:** 2016-01-27

**Authors:** Charles Elliott, Ram Gurajala, Craig Lisicki, Eunice Moon, Karunakaravel Karuppasamy

**Affiliations:** 1 School of Medicine, Case Western Reserve University, Cleveland, OH USA; 2 Radiology, Cleveland Clinic, Cleveland, OH USA

## Background

Patients who are being evaluated to undergo fibula free flap transfer often do not have arterial disease. However a significant anatomical variant cannot be clinically excluded. Contrast enhanced magnetic resonance angiogram (CE-MRA) is commonly performed to identify the suitable side to harvest the flap and to exclude unsuitable infra-popliteal arterial anatomy. The aim of this study is to measure the quality and accuracy of a non-contrast MRA (NC-MRA) technique (Native SPACE = Non-contrast Angiography of the Arteries and Veins using Sampling Perfection with Application Optimized Contrast by using different flip angle Evolution) compared to CE-MRA.

## Methods

Institutional review board approval was obtained for this study. Between October 2012 and August 2014, 16 patients underwent NC-MRA followed by CE-MRA to identify infra-popliteal arterial anatomy prior to fibular-free flap surgery. Clinically, none had symptoms of peripheral vascular disease. CE-MRA was performed in early and later arterial phases. After acquisition of one NC-MRA, it was repeated with different trigger delay at the discretion of the technologist to improve the quality. In this study, NC- and CE-MRA were randomly reviewed and their qualities were recorded on a 3- point scale for each leg: optimal (arteries and any significant disease are clearly detectable), suboptimal (arteries are detectable; however any significant disease cannot be excluded) and non-diagnostic (arteries are not detectable). Using CE-MRA as gold standard, sensitivity, specificity, positive and negative predictive values of NC-MRA in identifying the type of infra-popliteal arterial anatomy and significant disease (occlusion or >50% stenosis) was calculated.

## Results

Compared to 2-phase CE-MRA, an average 1.94 (SD 0.97) number of NC-MRA acquisitions were performed per patient. CE-MRA was optimal in all the legs (n = 32) and NC-MRA was optimal in 75%. NC-MRA was suboptimal in 19% and non-diagnostic in 6%. NC-MRA correctly identified normal and variant anatomy in 100% of optimal and suboptimal groups. Among the optimal quality NC-MRA (12 patients/69 infra-popliteal arteries), 58% demonstrated no significant disease. Compared with CE-MRA, the sensitivity, specificity, positive and negative predictive values of NC-MRA in identifying significant disease was 100%, 89%, 30% and 100% respectively.

## Conclusions

In patients undergoing MRA prior to fibular-free flap harvest, NC-MRA often provides optimal quality images of infra-popliteal arteries with high negative predictive value for significant disease. Administration of contrast to acquire CE-MRA should be reserved for those with suboptimal or non-diagnostic NC-MRA and when disease is suspected in an optimal quality NC-MRA.

**Figure 1:**
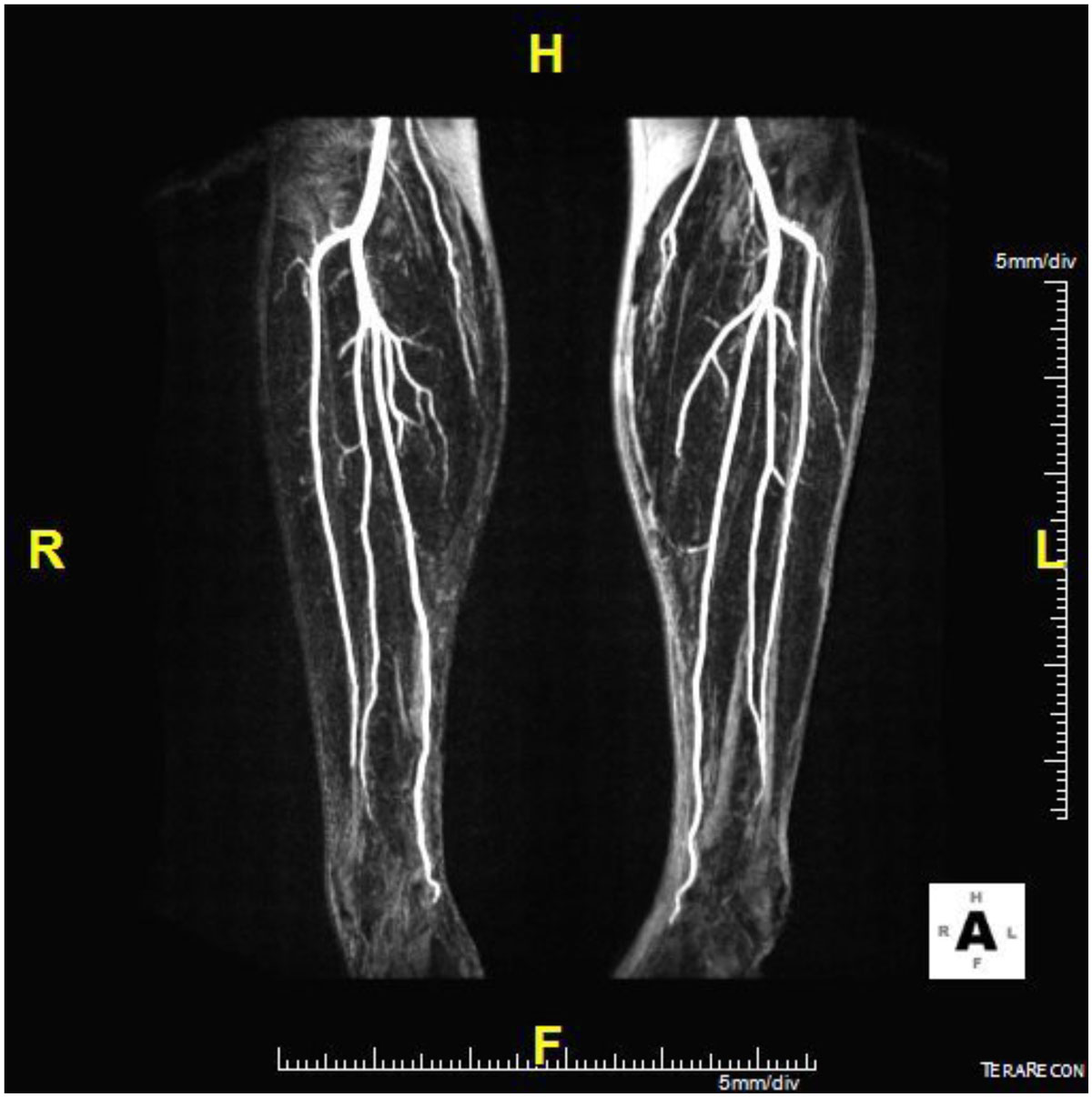
**Maximum intensity projection image of the subtracted data of two CE-MRA sequences: One obtained before and one obtained after adminstration of Gadolinium based contrast agent timed to arterial phase**.

**Figure 2:**
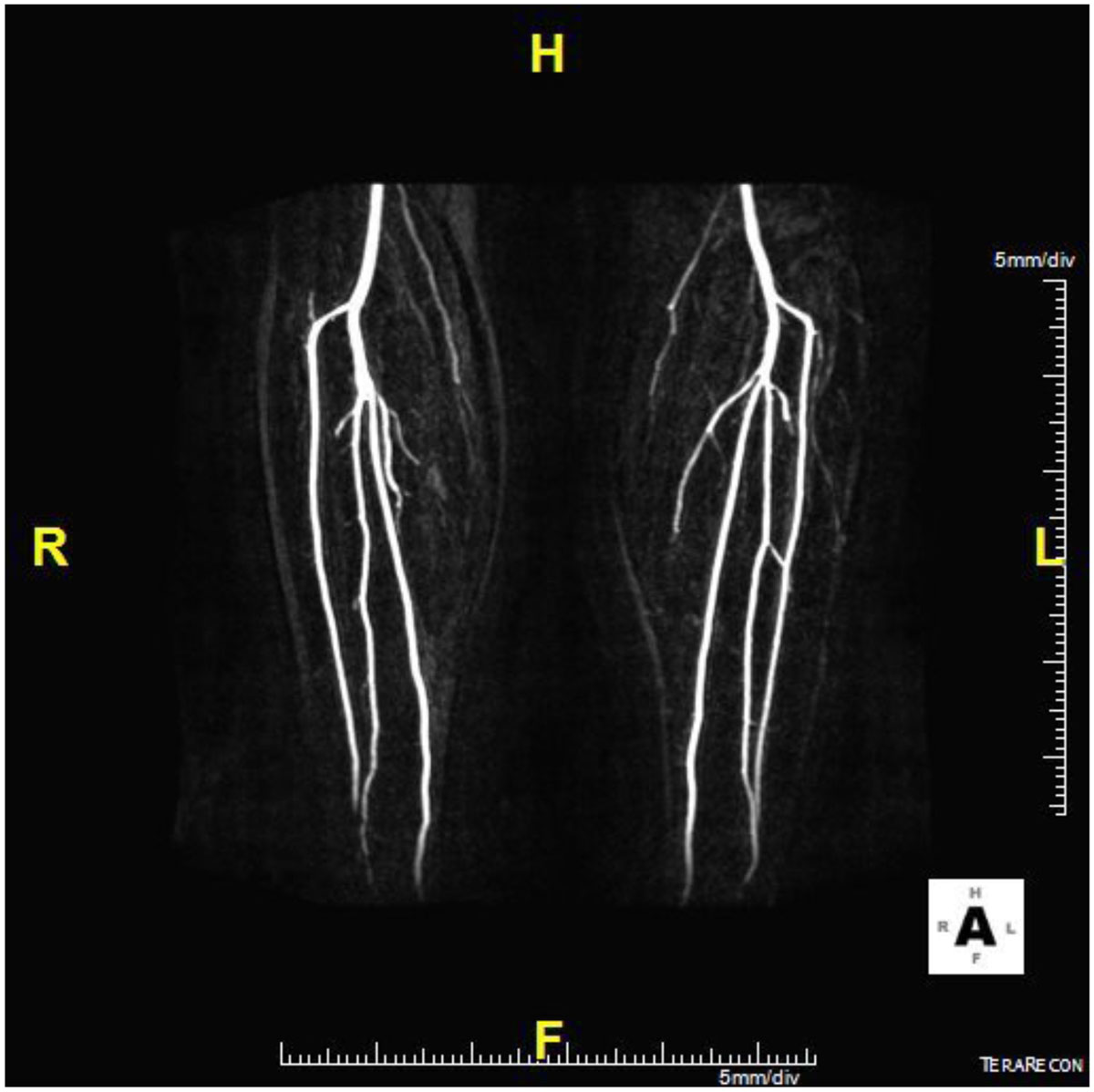
**Maximum intensity projection image of the subtracted data of two acqusitions within a Native SPACE sequence obtained when blood flow is minimal and maximal using different ECG trigger times**.

